# Characterization of the SARS-CoV-2 coronavirus X4-like accessory protein

**DOI:** 10.1186/s43042-021-00160-1

**Published:** 2021-05-08

**Authors:** Olanrewaju Ayodeji Durojaye, Nkwachukwu Oziamara Okoro, Arome Solomon Odiba

**Affiliations:** 1 Department of Molecular and Cell Biology, University of Science and Technology of China, Hefei, People’s Republic of China; 2 Guangxi Bioscience and Technology Research Centre, Guangxi Academy of Sciences, Nanning, 530007 People’s Republic of China; 3 Department of Pharmaceutical and Medicinal Chemistry, Faculty of Pharmaceutical Sciences, University of Nigeria, Nsukka, 410001 Nigeria; 4 Department of Molecular Genetics and Biotechnology, Faculty of Biological Sciences, University of Nigeria, Nsukka, Enugu State 410001 Nigeria; 5 Department of Biochemistry, College of Life Science and Technology, Guangxi University, Nanning, 530007 People’s Republic of China

**Keywords:** Coronavirus, COVID-19, SARS-CoV-2, X4 protein

## Abstract

**Background:**

The novel coronavirus SARS-CoV-2 is currently a global threat to health and economies. Therapeutics and vaccines are in rapid development; however, none of these therapeutics are considered as absolute cure, and the potential to mutate makes it necessary to find therapeutics that target a highly conserved regions of the viral structure.

**Results:**

In this study, we characterized an essential but poorly understood coronavirus accessory X4 protein, a core and stable component of the SARS-CoV family. Sequence analysis shows a conserved ~ 90% identity between the SARS-CoV-2 and previously characterized X4 protein in the database. QMEAN *Z* score of the model protein shows a value of around 0.5, within the acceptable range 0–1. A MolProbity score of 2.96 was obtained for the model protein and indicates a good quality model. The model has Ramachandran values of φ = − 57^o^ and ψ = − 47^o^ for α-helices and values of φ = − 130^o^ and ψ = + 140^o^ for twisted sheets.

**Conclusions:**

The protein data obtained from this study provides robust information for further in vitro and in vivo experiment, targeted at devising therapeutics against the virus. Phylogenetic analysis further supports previous evidence that the SARS-CoV-2 is positioned with the SL-CoVZC45, BtRs-BetaCoV/YN2018B and the RS4231 Bat SARS-like corona viruses.

## Background

World Health Organization (WHO) declared the novel coronavirus 2019-nCoV previously referred to as Wuhan-Hu-1, and now officially named SARS-CoV-2 the cause of the COVID-19 outbreak a public health emergency of international concern in January, 2020 [1, 2]. COVID-19 has become a major threat to health and economies around the world. More so, a second wave of spikes has been recorded across Europe, USA, and South America recently. Since the isolation of SARS-CoV-2 in 2019, laboratories have been in the race for therapeutics and vaccines in many countries [3, 4]. This race has yielded many drugs currently with Emergency Use Authorization (EUA) status including remdesivir [5], dexamethasone, convalescent plasma, and monoclonal antibodies (MABs). Several vaccine candidates are in the final stages of clinical trials from pharmaceutical companies including Johnson & Johnson, Novavax (NVAX), AstraZeneca’s (AZN), Moderna (MRNA), and Pfizer (PFE). Two of these pharmaceutical companies, Pfizer (PFE) and Moderna (MRNA), recently announced their vaccines to the over 90% and 94.5% safe and are currently being administered under EUA. So far, none of the current therapeutics in use, or vaccine candidates, has been certified to be an absolute cure. One of the major reasons amongst many of the possible causes for this setback may be based on very recent evidence that the coronavirus undergoes quick mutation in its genome [6], as strains genetically different from the originally sequenced strain have been isolated. Tackling this challenge will require targeting a highly conserved and stable region of the virus core structure as the bedrock for the design of new therapeutics.

Viruses have a relatively small genome and usually need a host to suitable execute their life cycle. The *Coronaviridae* have a genome spanning 26 to 32 kb positive-sense RNA [7–9]. Coronaviruses (CoVs) like the severe acute respiratory syndrome (SARS) and Middle-East respiratory syndrome (MERS) viruses are primarily zoonotic [10]. Humans are a complex species in terms of genome; however, the human system is highly susceptible to this “respiratory-philic” pathogenic virus, which if untreated is fatal. These class of viruses have a conserved small integral membrane CoV envelope protein necessary for budding, packaging, envelope formation, as well as a contributing factor to its pathogenesis [9]. Understanding the biochemistry and molecular structure of this highly conserved structure is a major factor needed to kill the pathogen, as designing therapeutics is totally dependent on understanding the structural composition. Members of this group of coronaviruses have four structural proteins namely, membrane (M), spike (S), nucleocapsid (N), and envelope (E) [11]. They also have the X4 (ORF7a) accessory proteins, but their functions are still not yet well understood. The coronavirus X4 protein is vital to the survival and replication of the coronavirus as recent studies show that X4 is involved during the replication cycle of the SARS-CoV [12]. Targeting this protein with suitable binding moieties that could interrupt the function of this protein may support other existing strategies to treat this infection. In this study, we did not focus on targeting the X4 protein rather, we characterized molecular the structure of the SARS-CoV-2 X4 protein, alongside some predicted biochemical features as a bedrock for further studies; providing valuable information for the design of therapeutics. We also further compared it with other homologues in other species as supportive evidence for its lineage amongst the *Coronaviridae*.

## Methods

### Sequence data and alignment

The genome sequence data of the isolated SARS-CoV-2virus was sourced from the GenBank database (MN908947.3, which has 100% homology with NC_045512.2). We considered the nucleotide sequence between 26,683 and 29,903 as the region within which to find the location of the X4 protein, since based on previous studies, the X4 sequence is located in this region coding for several of the accessory proteins. EMBOSS transeq and backtranseq were used for sequence translation and back translation, respectively [13]. Clustal Omega software package was used for all alignments between SARS corona virus X4 protein and SARS-CoV-2 [14]. Within this sequence, we found a portion of the 83 amino acid residues with homology to the SARS corona virus X4 protein, and it is the sequence of interest for further studies.

### Homology modeling

The homology modeling of the SARS-CoV-2 aligned segment was done using the SWISS-MODEL (http://swissmodel.expasy.org) for automated comparative modeling of three-dimensional (3D) protein structures [15]. QMEAN (Qualitative Model Energy Analysis) was used for the assessment of the model protein quality [16]. A considerable number of alternative models were produced, from which subsequently the final model was selected based on produced scores. We employed MolProbity (version 4.4) to evaluate the model global and local protein quality [17–19], and Ramachandran plot for torsion angles between residues. In sequence order, φ is the N(i − 1), C(i), Ca(i), N(i) torsion angle and ψ is the C(i), Ca(i), N(i), C(i + 1) torsion angle. The φ values were plotted on the *x*-axis while the ψ values on *y*-axis.

### 3D structure comparison

The 3D modeling of the SARS-CoV-2 genome translated segment was followed by a structural comparison with the X4 protein 3D structure (PDB: 1YO4) using the UCSF Chimera [20]. High-quality images were generated and presented using amino Pymol molecular visualizer [21].

### Protein physiochemical parameters

Calculation of the physiochemical parameters of proteins is a sub-function of the ExPASy server, basically for protein identification, and was used for determining the physiochemical parameters such as theoretical isoelectric point, molecular weight, amino acid composition, extinction coefficient, and instability index [22].

### Phylogenetic analysis

We employed Tamura-Nei model for phylogenetic analysis and is based on the maximum likelihood using MEGA5 program [23].

## Results

**Fig. 1:**
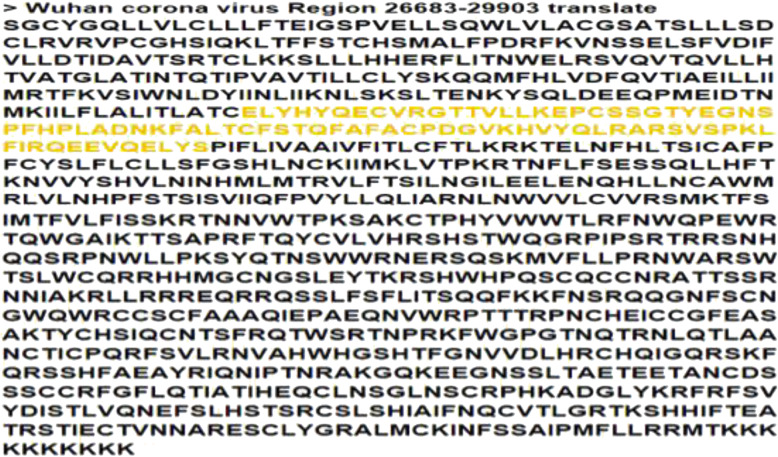
Translated sequence of the SARS-CoV-2 corona virus nucleotide sequence with the highlighted segment forming the model protein coding sequence of interest

**Fig. 2:**
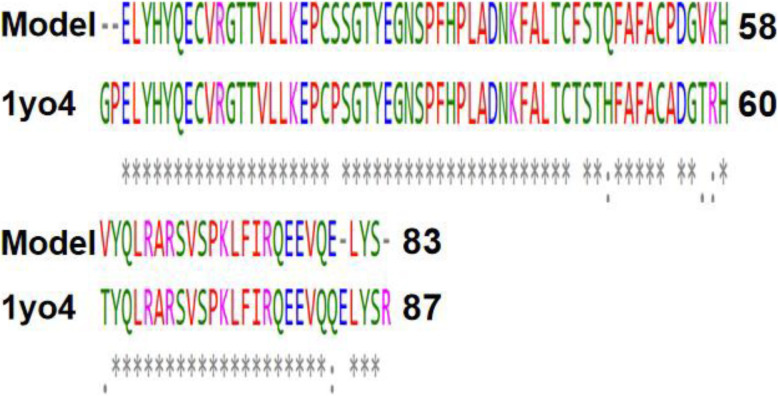
Sequence alignment between the amino acid sequence of the model protein and the SARS related corona virus X4 protein. As depicted, few homology differences were noticed. Single asterisk (*) represents regions with complete conservation, while colon (:) represents conservation between amino acid residues with similar properties. Period (.) represents conservation between amino acids with less similar properties. The non-conserved regions are empty space

**Fig. 3:**
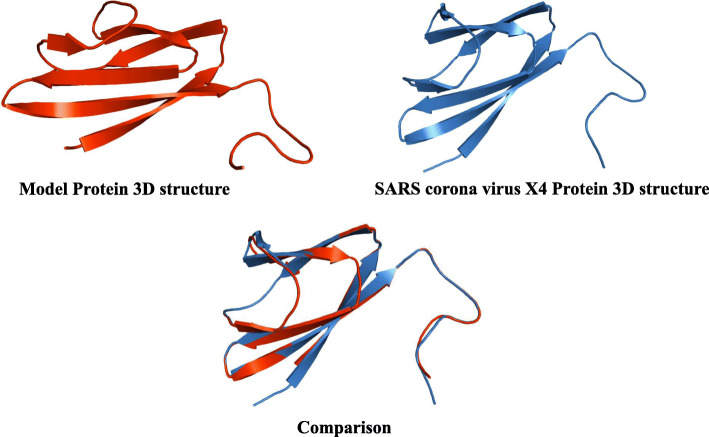
3D structures of the model and template protein with the structural comparison. Model protein is presented in red while the template in blue. The matching together of the two was depicted in the mixed picture beneath for comparison

The full genome of the SARS-CoV-2 consists of 29,903 nucleotides but here, nucleotides between 26,683 and 29,903 were considered as the portion coding for the group of proteins from which we intended to find the particular protein of our interest, and direct translation of this segment of nucleotides produced a sequence of 1004 amino acids after the deletion of existing stop codons (Fig. 1). The deletion of stop codons was necessary as the 3D homology tool used for the modeling of the reference protein of interest does not recognize them. We used the highlighted segment in Figs. 1 and 2 for the predicted 3D structure modeling in comparison with the X4 protein 3D structure (Fig. 3).

**Fig. 4:**
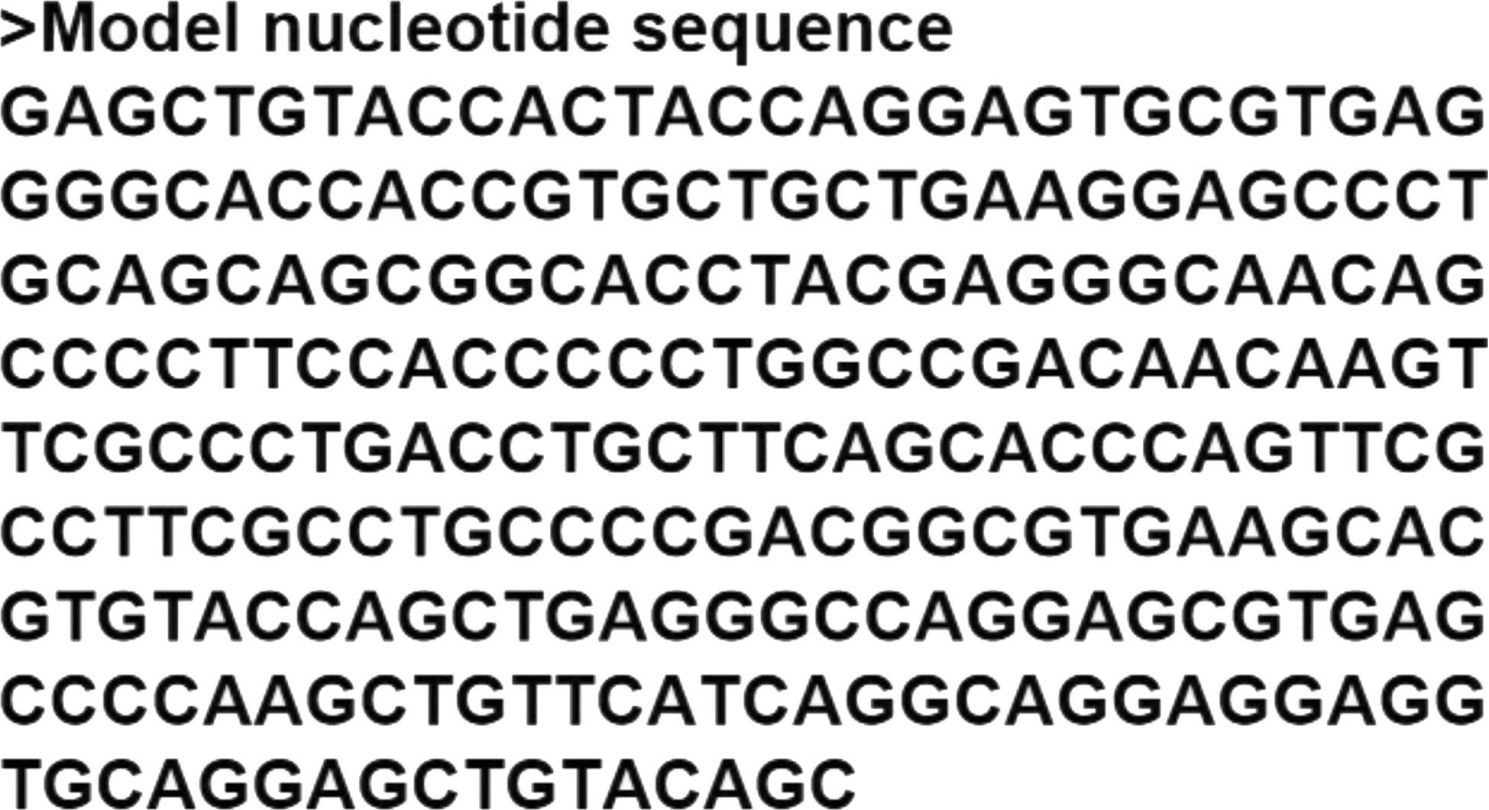
Back-translation of the model protein amino acid sequence to generate the corresponding nucleotide sequence

**Fig. 5:**
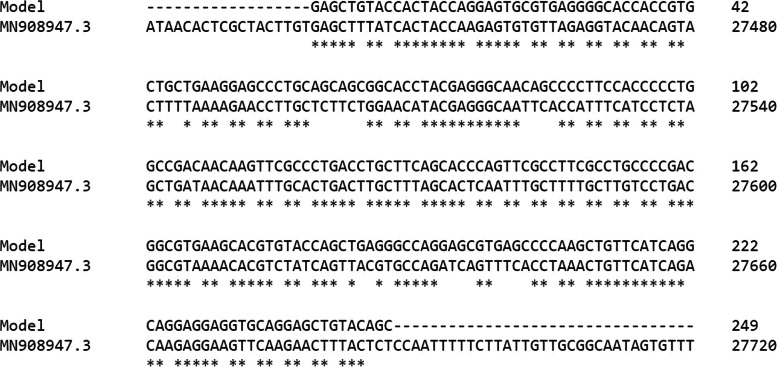
Sequence alignment between the model protein nucleotides and the 27,439 to 27,684 nucleotide region of the SARS-CoV-2 complete genome. Single asterisk (*) represents regions with complete conservation, while colon (:) represents conservation between amino acid residues with similar properties. Period (.) represents conservation between amino acids with less similar properties. The non-conserved regions are empty space

The amino acid sequence of the model protein was back-translated (Fig. 4) to generate the corresponding nucleotide sequence which was then aligned with the SARS-CoV-2 full genome (Fig. 5). This back-translated sequence alignment shows that the homology between the model protein sequence and the SARS-CoV-2 complete genome is located between 27,439 and 27,684.

**Fig. 6:**
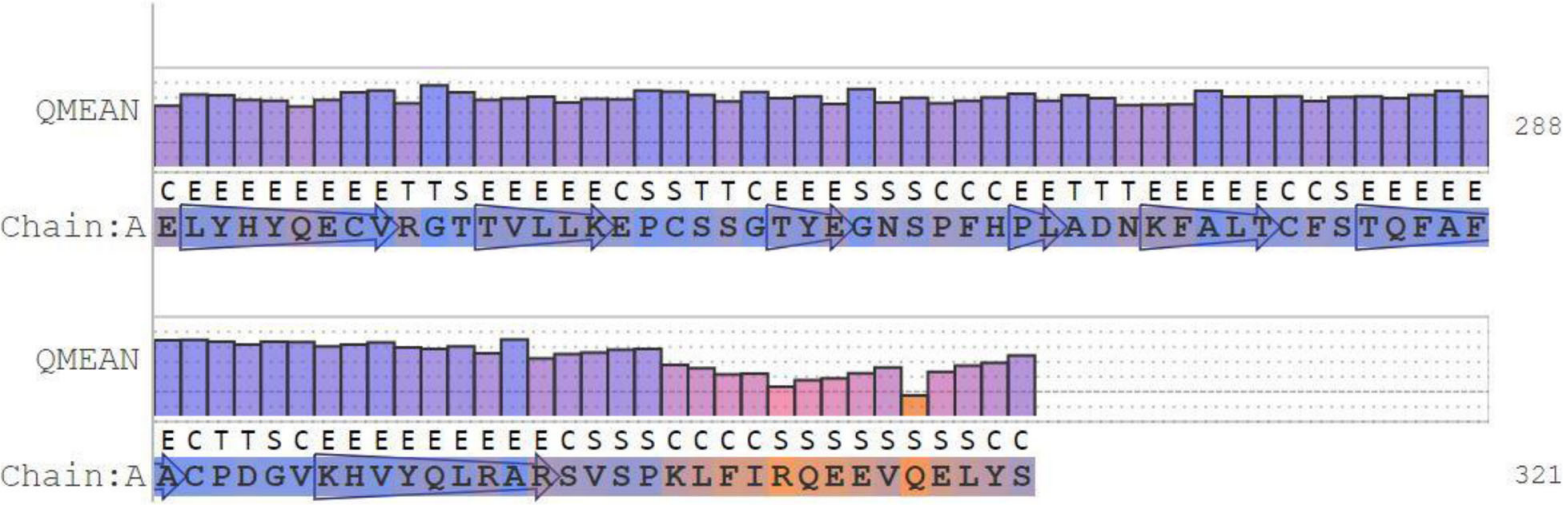
Residue quality chart which depicts the absolute quality of the model protein on the basis of individual amino acid residue

**Fig. 7:**
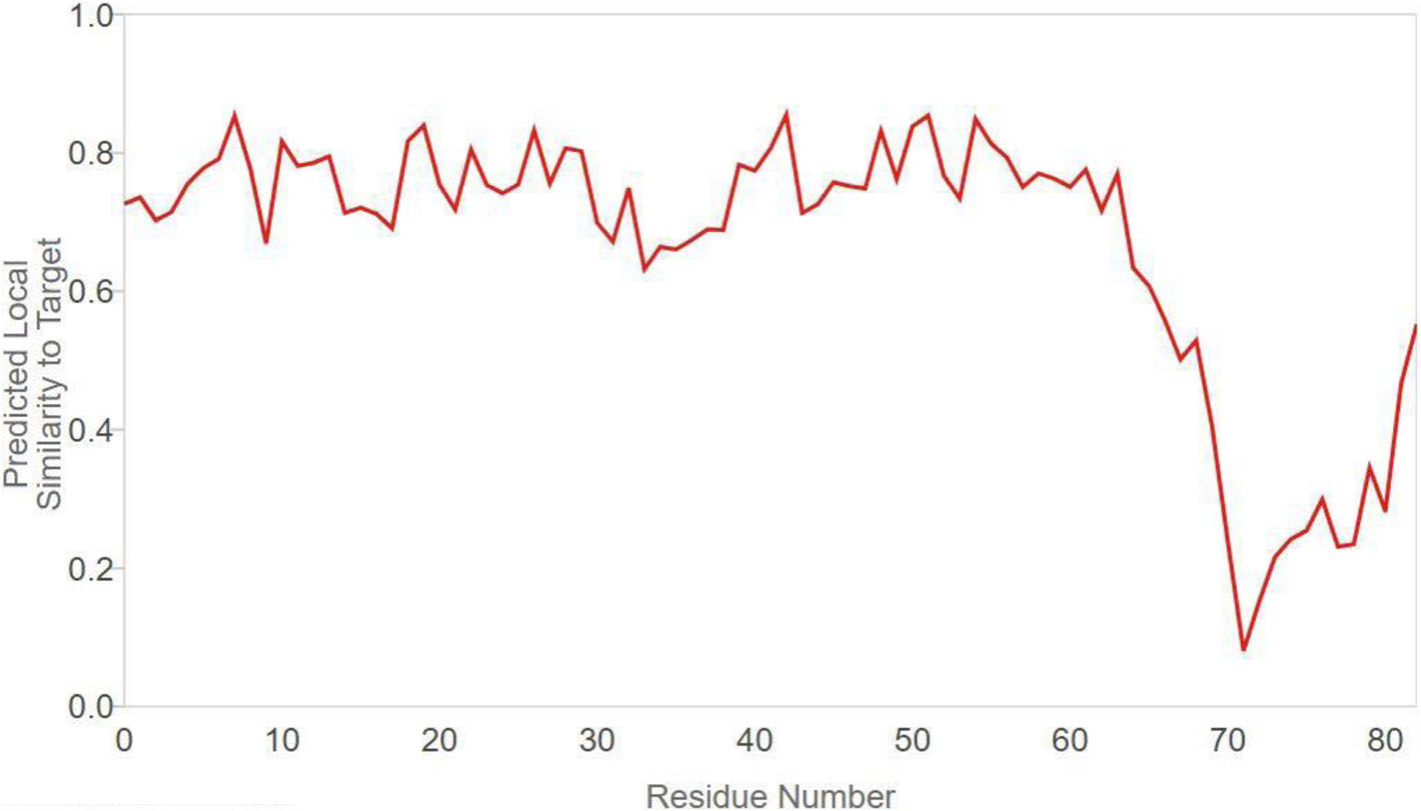
Local quality estimate graph showing the values of the predicted local similarity to target plotted against the model protein residue number

**Fig. 8:**
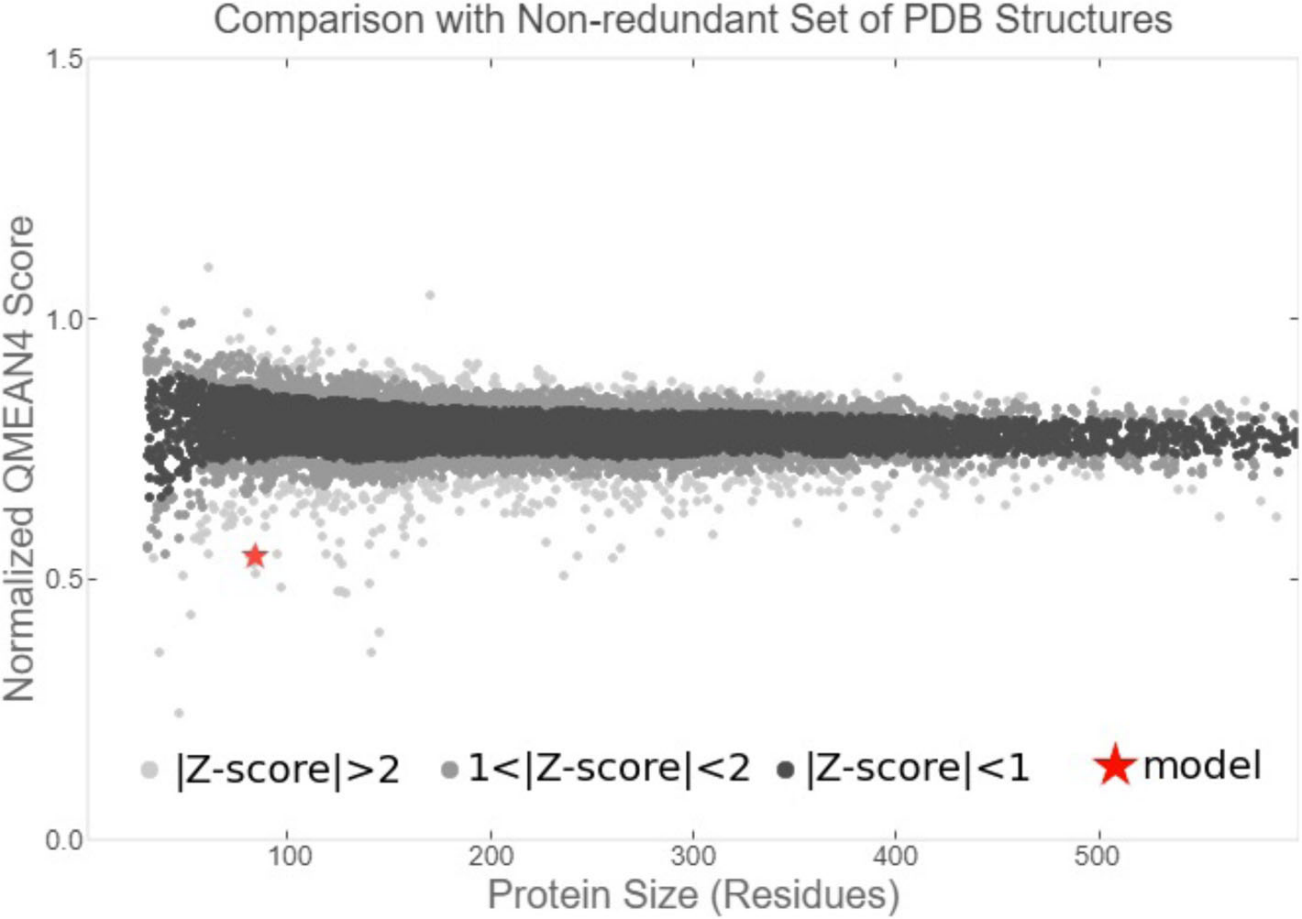
Graphical presentation of estimation of absolute quality of the model protein

The result of the QMEAN parameter scores of the model protein based on the composite scoring function (which evaluates several structural features of the model protein) are presented in Figs. 6, 7, and 8 and Table 1. The absolute quality estimate of the model is expressed in terms of how well the model score agrees with the expected values from a representative set of high-resolution experimental structures (Fig. 6). There are two global score values, QMEAN4 (for linear combination of statistical potential) and QMEAN6 (assessing prediction-based consistency of structural features). Both global scores are originally in a range [0,1] with one being good. By default they are transformed into *Z* scores to relate them with what we would expect from high resolution X-ray structures. The local scores are a linear combinations of the 4 statistical potential terms as well as the agreement terms evaluated on a per residue basis. They are as well in the range [0,1] with one being good (Fig. 7).

**Table 1:** *Z* score for the individual components of QMEAN for the model protein

Components	Scores
QMEAN score	− 4.18
Interaction energy of C_β	− 1.22
Pairwise energy of all atoms	− 1.31
Solvation energy	− 1.30
Torsion angle energy	− 3.47

When compared to the set of non-redundant protein structures, the QMEAN ***Z*** scores as shown in Fig. 8 were close to 0. Good models have scores < 1 and are often located in the dark zone.

**Fig. 9:**
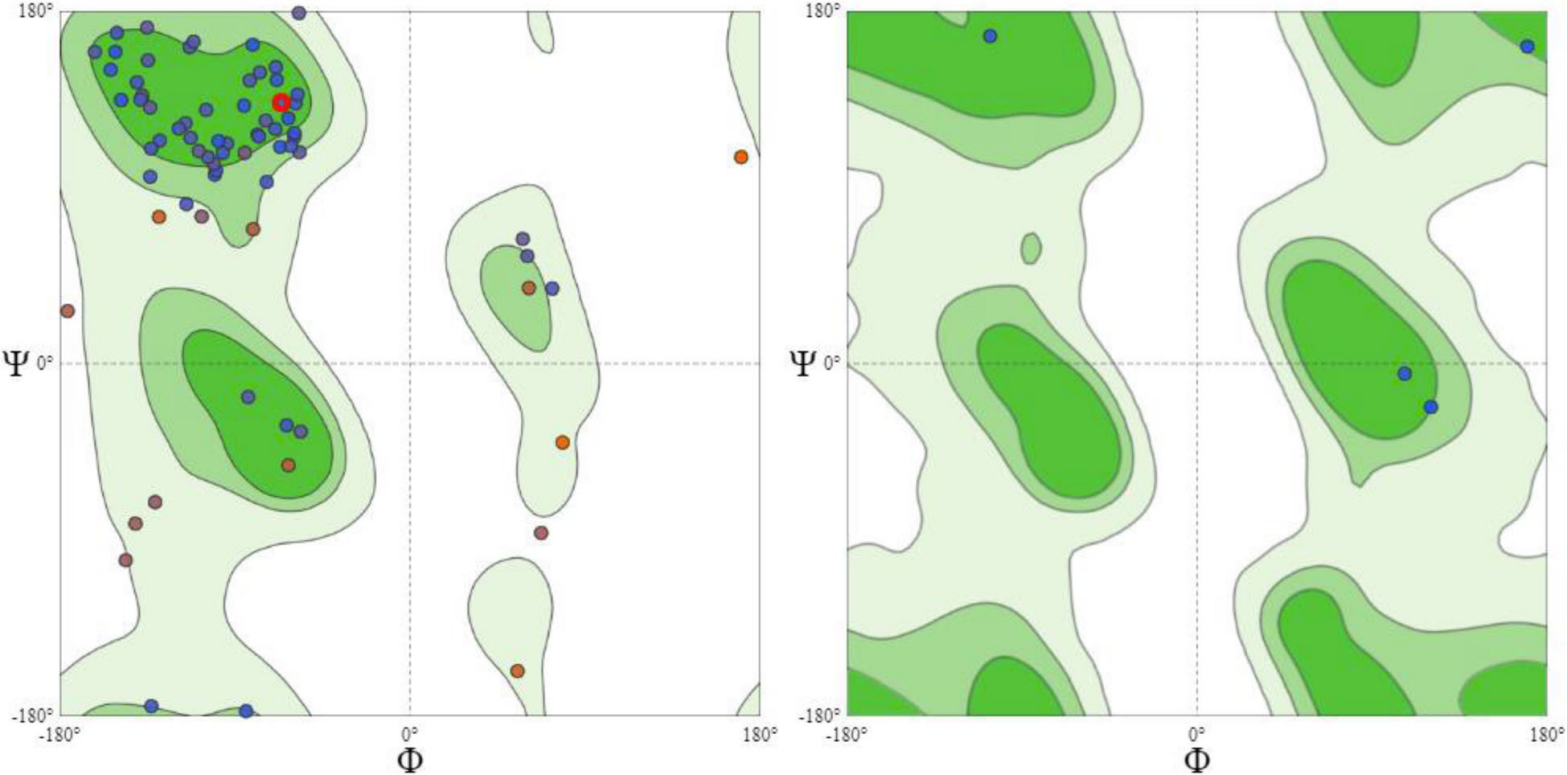
Presented here are two Ramachandran plots. The plot on the left hand side is hand side shows the general torsion angles for all the residues in the model protein while the plot on the right hand side is specific for the glycine residues of the protein

**Fig 10:**
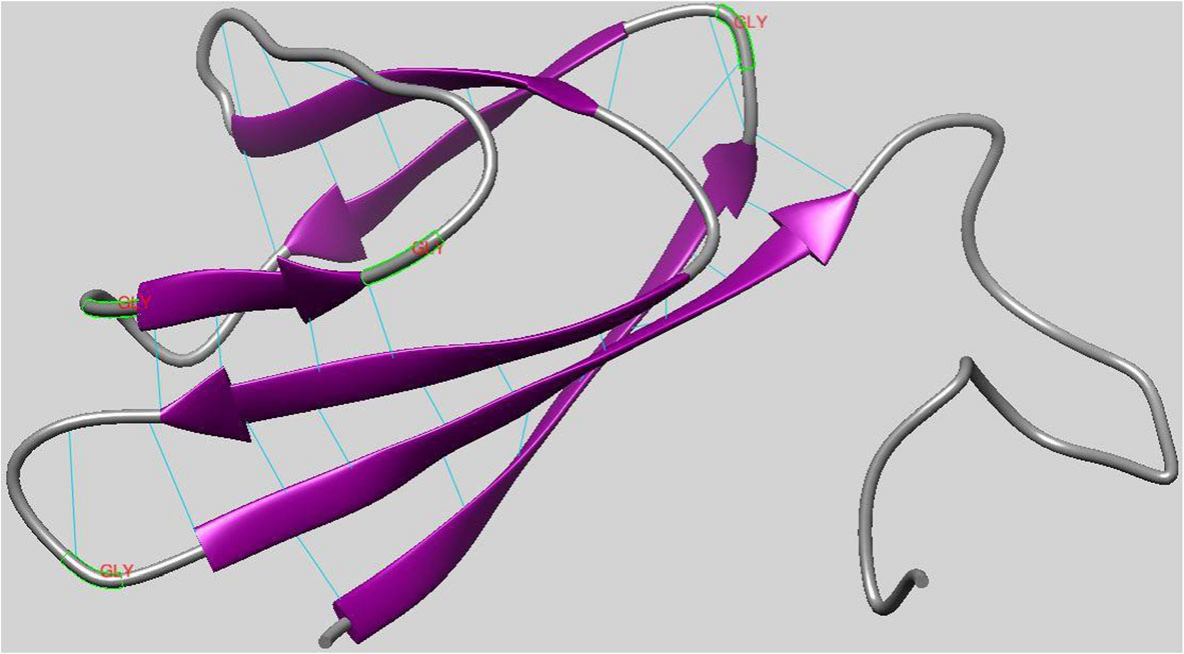
The model protein secondary structures with the inter model hydrogen bonds. Regions of beta sheets and loops are shown in purple and grey colors, respectively. Labeled in red are the glycine residues of the loops

The restriction of the Ramachandran angles in the protein to certain values is visible in the Ramachandran plot in (Fig. 9). The plot shows that each type of secondary structure elements occupies its characteristic range of φ and ψ angles. The horizontal axis shows φ values, while the vertical shows ψ values. Each dot on the plot shows the angles for an amino acid. The counting starts in the left hand corner from − 180 and extend to + 180 for both the vertical and horizontal axis. This is a convenient presentation and allows clear distinction of the characteristic regions of α-helices and β-sheets. An exception from the principle of clustering around the α- and β-regions can be seen on the right plot of Fig. 9. In this case, the Ramachandran plot shows torsion angle distribution for one single residue, glycine. Glycine does not have a side chain, which allows high flexibility in the polypeptide chain, making forbidden rotation angles accessible. This explains why glycine is often found in loop regions, where the polypeptide chain needs to make a sharp turn. This is further depicted in the model protein secondary structures (Fig. 10). Model and template protein comparative physiochemical parameters ProtParam were obtained from the amino acid sequences of the individual proteins (Tables 3 and 4).

**Fig. 11:**
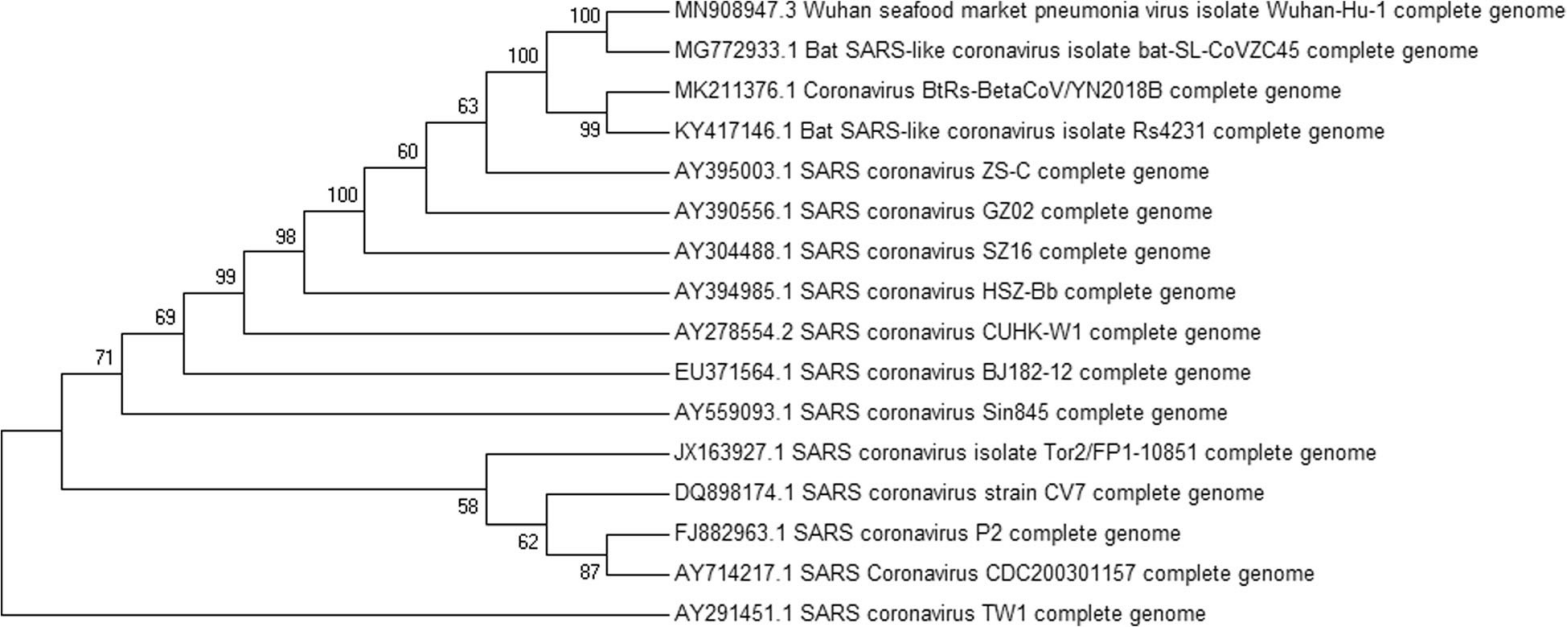
Bootstrap consensus phylogenetic tree based on the model protein sequence

The phylogeny tree with the highest log likelihood (**−** 80762.5778) based on the model protein sequence is shown in Fig. 11. The percentage of trees in which the associated taxa clustered together is shown next to the branches as conducted in MEGA5.

## Discussion

Proteins that share a high sequence similarity are likely to have very similar three-dimensional structures and by implication similar function [24, 25]. In this study, the target protein was modeled using the SARS-CoV protein X4 as template. This selection was based on the high resolution and its identity with the target protein which is as high as 91.57%. The SARS-CoV-2 nucleotides between 26,683 and 29,903 were considered as the portion coding for a group of protein, of which our target protein of interest is found, and directly translated to produce a sequence of 1004 amino acids (Fig. 1). Structural differences were noticed when alignment analysis was carried out on the sequence (Figs. 2 and 3). The percentage amino acid sequence identity between the model and the template protein shows a high level of conservation, with 90% identity observed between both sequences, showing that the conserved domains are predominant. Also, the alignment between the back-translated model protein nucleotides and the 27,439 to 27,684 nucleotide portion of the SARS-CoV-2 complete genome shows that the model protein coding sequence is located between 27,439 and 27,684 nucleotides of the viral genome (Figs. 4 and 5).

The absolute quality estimate of the model is expressed in terms of how well the model score agrees with the expected values from a representative set of high-resolution experimental structures (Fig. 6). The QMEAN scores were transformed into *Z* scores to decipher the model of a high resolution X-ray structure, and the values are within range (Fig. 7). Our study shows the *Z* score of the model protein has a value of around 0.5, which falls within the acceptable range 0–1, as indicated in Fig. 8 and such a score is an indication of a relatively good model as it is close to zero which is the average *Z* score for a good model [26]. Lower MolProbity (MP scores) clash score values are expected to be an indication of good models as proven by the clash score value  (Table 2) exhibited by the experimental protein that was modeled for the purpose of this study [17–19]. Rotamer outliers asymptote to a value of < 1% at high resolution, a general-case Ramachandran outliers to < 0.05%, and Ramachandran favored to 98%. With a 3.07 clash score, and a 76.54% Ramachandran favored region value as compared to the Ramachandran outliers and rotamer outliers individual values of 4.94% and 27.03%, respectively, we arrived at a MolProbity score of 2.96. This value is low enough to indicate the quality of a good model in the experimental protein [17].

**Table 2:** The individual parameters and scores as calculated by MolProbity

Parameters	Scores
MolProbity score	2.96
Clash score	3.07
Ramachandran favored	76.54%
Ramachandran outliers	4.94%
Rotamer outliers	27.03%

The repetitive nature of secondary structures is due to the repetitive conformation of the residues and, ultimately, repetitive values of φ and ψ. The varied secondary conformations can be differentiated by their φ and ψ values with the values of different secondary conformations mapping to different areas of the Ramachandran plot [27]. The Ramachandran plot peptides have points clustered about the values of φ = − 57^o^ and ψ = − 47^o^ which are the average values for α-helices while the plot for twisted beta sheets have points clustered about the values of φ = − 130^o^ and ψ = + 140^o^ which are the average values for twisted sheets. The core regions (green in Fig. 9) contain the most favorable combinations of φ and ψ and contain the greatest number of points. The result also shows a small third core region in the upper right quadrant. This is called the allowed region and can be situated around the core regions or unassociated with a core region and it contains fewer data points than the core regions [27]. The remaining areas of the plot are considered disallowed. Since glycine residues have only one hydrogen as side chain and has φ and ψ values of + 55^o^ and− 116^o^, respectively which does not exhibit steric hindrance and for that reason positioned in the disallowed region of the Ramachandran plot as shown in the right hand side plot (Fig. 9). The extinction coefficient reveals how much light a protein absorbs at a certain wavelength. It is useful to have an estimation of this coefficient for monitoring a protein in a spectrophotometer when purifying it, and estimating the molar extinction coefficient determined from the amino acid composition [28] which is shown in Table 3.

**Table 3:** Amino acid composition table for both the template and model proteins

	Amino acid residues in one letter codes																			
Proteins	A	R	N	D	C	Q	E	G	H	I	L	K	M	F	P	S	T	W	Y	V
**Template**	6	6	2	2	4	5	7	5	4	1	8	3	0	5	6	6	8	0	5	4
**Model**	5	4	2	2	4	5	7	4	3	1	8	4	0	6	5	7	5	0	5	6

It has been shown that the identity of the N-terminal residue of a protein plays an important role in determining its stability in vivo [29–32]. A protein with instability index smaller than 40 is predicted as stable; and above 40 is considered unstable [33, 34]. The comparative instability index values for the template and model proteins were 66.61 and 56.58, respectively, showing both are unstable proteins. A protein’s aliphatic index is the relative volume occupied by aliphatic side chains (isoleucine, alanine, leucine, and valine). It may be regarded as an indication for the increase in thermostability of globular proteins. The aliphatic index of the experimental proteins were calculated according to the following formula [35].

Aliphatic index = X(Ala) + *a* × *X*(Val) + *b* × [X(Ile) + X(Leu)]

where *X*(Ala), *X*(Val), *X*(Ile), and *X*(Leu) are mole percent (100 × mole fraction) of alanine, valine, isoleucine, and leucine. The “*a*” and “*b*” coefficients are the relative volume of valine side chain with a value of *a* = 2.9 and of Leu/Ile side chains *b* = 3.9 to the side chain of alanine. The aliphatic index calculated for the experimental protein shows a higher thermostability for the model protein than the template.

It has been shown that α-helices are more stable, robust to mutations and designable than β-strands in natural proteins [36]. The template and model proteins respectively have a total of 87 and 83 amino acid residues (Table 4) with the composition of individual residues shown in Table 3. As shown in Fig. 10, the model protein which shares a structural homology with the template is predominantly occupied by residues forming beta sheets and coils, with none forming helices. The instability observed for these two proteins from their physiochemical characteristics show that the unavailability of residues forming alpha helix may be the accountable factor. In this study, we also compared a genome of interest to similar genomes in the GenBank database to predict the evolutionary relationships between homologous genes represented in the genomes of each divergent species [8, 23, 24]. Organisms with common ancestors were positioned in the same monophyletic group in the tree and the same clade where the genome of interest (SARS-CoV-2) is positioned with the SL-CoVZC45, BtRs-BetaCoV/YN2018B, and the RS4231, all which are Bat SARS-like corona viruses [37]. This shows that the four viral strains share a common source with shorter divergence period. TW1 virus, a SARS corona virus is the most distantly related based on its branch length and as such can be regarded as an outlier in the tree.

**Table 4:** Calculated physiochemical properties by the ExPASy ProtParam server

Calculated parameters	Template protein	Model protein
**Molecular weight**	9896.10	9478.71
**Theoretical** ***pI***	7.06	6.32
**Amino acid composition (total)**	87	83
**Atomic composition**	C_438_H_667_N_123_O_132_S_4_	C_426_H_644_N_112_O_126_S_4_
**Extinction coefficient**	7700	7700
**Estimated half-life**	30 h (mammalian reticulocytes, in vitro). > 20 h (yeast, in vivo). > 10 h (Escherichia coli, in vivo).	30 h (mammalian reticulocytes, in vitro). > 20 h (yeast, in vivo). > 10 h (Escherichia coli, in vivo).
**Instability index**	66.61	56.58
**Aliphatic index**	60.57	69.28
**GRAVY**	− 0.569	− 0.343

## Conclusions

We modeled the target protein using the hypothetical protein X4 as template based on a high similarity index of 91.57%, as revealed by sequence analysis where the percentage amino acid sequence identity between the model and the template protein shows a high level of conservation. The QMEAN value show that the model generated for study here is within the acceptable standard and amenable to structural analysis, including X-ray resolution. All the predicted structural parameters for this model protein studied such as the MolProbity (MP scores) clash score, staggered χ angles, Ramachandran values (φ and ψ), all demonstrate a protein that is suitable for further study and a potential target for therapeutics and vaccines. However, the comparative instability index values for the template and model proteins were 66.61 and 56.58, respectively, suggesting that the protein may be too sensitive for in vitro studies. On the other hand, the aliphatic index shows that the thermostability of the model protein is higher than the template and may withstand more harsh conditions during experimental studies. Our results supporting previous studies, show that the SARS-CoV-2is positioned with other Bat SARS-like corona viruses including SL-CoVZC45, BtRs-BetaCoV/YN2018B, and the RS4231.

## Data Availability

Data are available on the appropriate databases cited.

## Abbreviations

SARS-CoV-2: Severe acute respiratory syndrome coronavirus 2
2019-nCoV: 2019 novel coronavirus
SARS-CoV: Severe acute respiratory syndrome coronavirus
MERS: Middle East respiratory syndrome
SARS: Severe acute respiratory syndrome
pI: Isoelectric point
DB: Protein Data Bank
ARD: Acute respiratory disease
DNA: Deoxyribonucleic acid
RNA: Ribonucleic acid
hCoV: Human coronavirus
NCBI: National Center for Biotechnology Information
INSDC: International Nucleotide Sequence Database Collaboration
QSQE: Quaternary structure quality estimate
CFSSP: Chou and Fasman Secondary Structure Prediction
MEGA: Molecular Evolutionary and Genetics Analysis
QMEAN: Qualitative Model Energy Analysis
GRAVY: Grand average of hydropathy
